# Urban habitat fragmentation and floral resources shape the occurrence of gut parasites in two bumblebee species

**DOI:** 10.1002/ece3.10299

**Published:** 2023-07-12

**Authors:** Nicola Tommasi, Beatrice Colombo, Emiliano Pioltelli, Paolo Biella, Maurizio Casiraghi, Andrea Galimberti

**Affiliations:** ^1^ ZooplantLab, Department of Biotechnology and Biosciences University of Milano‐Bicocca Milan Italy; ^2^ NBFC, National Biodiversity Future Center Palermo Italy

**Keywords:** beekeeping, bee‐parasites interaction, bumblebees, habitat loss and fragmentation, landscape epidemiology, urbanization

## Abstract

Urbanization and the expansion of human activities foster radical ecosystem changes with cascading effects also involving host‐pathogen interactions. Urban pollinator insects face several stressors related to landscape and local scale features such as green habitat loss, fragmentation and availability reduction of floral resources with unpredictable effects on parasite transmission. Furthermore, beekeeping may contribute to the spread of parasites to wild pollinators by increasing the number of parasite hosts. Here we used DNA‐based diagnostics tools to evaluate how the occurrence of parasites, namely microsporidians (*Nosema* spp.), trypanosomatids (*Crithidia* spp.) and neogregarines (*Apicystis bombi*), is shaped by the above‐mentioned stressors in two bumblebee species (i.e. *Bombus terrestris* and *Bombus pascuorum*). Infection rates of the two species were different and generally higher in *B. terrestris*. Moreover, they showed different responses towards the same ecological variables, possibly due to differences in body size and foraging habits supposed to affect their susceptibility to parasite infection. The probability of infection was found to be reduced in *B. pascuorum* by green habitat fragmentation, while increased along with floral resource availability. Unexpectedly, *B. terrestris* had a lower parasite richness nearby apiaries maybe due to the fact that parasites are prone to be transmitted among the most abundant species. Our finding supports the need to design proper conservation measures based on species‐specific knowledge, as suggested by the variation in the parasite occurrence of the two species. Moreover, conservation policies aiming at safeguarding pollinators through flower planting should consider the indirect effects of these measures for parasite transmission together with pollinator biodiversity issues.

## Graphical abstract

Urbanization and the expansion of human activities foster radical ecosystem changes with cascading effects also involving host‐pathogen interactions. Urban pollinator insects face several stressors related to landscape and local scale features such as green habitat loss, fragmentation and availability reduction of floral resources with unpredictable effects on parasite transmission. Furthermore, beekeeping may contribute to the spread of parasites to wild pollinators by increasing the number of parasite hosts. In *Bombus terrestris* and *Bombus pascuorum*, two bumblebee species, infection rates were different and generally higher in *B. terrestris*. The probability of infection was found to be reduced in *B. pascuorum* by green habitat fragmentation, while increased along with floral resource availability. Unexpectedly, *B. terrestris* had a lower parasite richness nearby apiaries probably because parasites are prone to be transmitted among the most abundant species. These results highlight that conservation policies aiming at safeguarding pollinators through flower planting should consider the indirect effects of these measures on parasite transmission together with pollinator biodiversity issues.

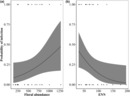

## 1 INTRODUCTION

Urbanization is growing rapidly worldwide leading to habitat loss and modification, with detrimental consequences for biodiversity and ecological functioning (Ayers & Rehan, 2021; Fisogni et al., 2020; Wenzel et al., 2020). Wild pollinator insect communities are deeply affected by this phenomenon, especially in the case of bees (Hymenoptera: Anthophila). While some species are able to successfully exploit these conditions and thrive in urban areas (Fitch et al., 2019), others are facing challenges due to the lack of nesting and foraging habitat (Cane, 2005), distance between green areas and hostile climatic conditions (e.g. urban heat islands) (Biella et al., 2022), pollution (Tommasi, Pioltelli, et al., 2022) or parasite infections (Youngsteadt et al., 2015). From a landscape perspective, urbanization significantly shapes land‐use features such as composition (the proportion of different land‐use categories) and configuration (the spatial arrangement of patches of land‐use categories) (Fu & Weng, 2016), with direct consequences for bee communities. In this context, green habitats act as islands which are exploited by wild bees, and whose loss has been associated with negative effects on bee species richness (Winfree et al., 2009) and their foraging habits (Andrieu et al., 2009; Tommasi, Biella, et al., 2022). Similarly, green habitat fragmentation may affect pollinator species foraging behaviour (Andrieu et al., 2009) and richness with stronger consequences for the smaller and less mobile species (Ayers & Rehan, 2021).

Urbanization may also have cascading effects on the health and fitness of wild bees, for example by shaping the dynamics of their parasite infections (Figueroa et al., 2020) contributing to the population decline of both managed and wild bee populations (Ivers et al., 2022). For instance, urban green habitat availability and fragmentation may indirectly affect parasite spread by shaping features of the host community such as species richness and abundance (Tommasi, Biella, et al., 2022; Tommasi, Pioltelli, et al., 2022). Indeed, previous research focused on bumblebees suggested that urbanization may promote the diffusion of these parasites (e.g. *Crithidia* spp. and *Nosema* spp.) in wild bees (Theodorou et al., 2016) also due to changes in bumblebee community features (Ivers et al., 2022), increasing the pressure experienced by these organisms in urban habitats (but see Samuelson et al., 2020). However, despite the increasingly higher research effort towards parasite spillover among bees, the role of landscape and local features of green habitat in shaping parasite dynamics is a topic largely neglected in the literature.

Inter and intraspecific transmission of parasites usually occurs through direct contact between hosts or via contamination of flowers visited by infected individuals (Cilia et al., 2023; Singh et al., 2010). In this context, a major role of beekeeping in contributing to the spread of parasites potentially infecting wild species has also been reported (Cilia et al., 2023; Dolezal et al., 2016; Meeus et al., 2011). Since apiculture exceptionally increases the number of potential hosts in the area surrounding honeybee hives, this anthropic activity could facilitate both the direct contact between honeybees and wild species and the contamination of floral resources. Beekeeping has gained growing importance worldwide, especially in urban areas (Matsuzawa & Kohsaka, 2021), due to its positive impact on community building and environmental education (Skelton, 2006). Although several studies showed the important effects of apiculture on parasite distribution in the wild pollinator community (Graystock, Blane, et al., 2016), nowadays dynamic and direct causes of this impact are still unclear (Cilia et al., 2023).

To investigate the role of environmental features on parasite occurrence different methodological approaches can be employed. For example, histopathological evaluation as well as species‐specific fluorescence in situ hybridization (FISH) can be employed for this purpose and have the advantages of being able to clearly localize the infections and also highlight the damages induced on tissues by parasites (Panek et al., 2018). Other methods such as those based on PCR can be efficient alternatives to more traditional approaches. Indeed, PCR‐based methodologies are rapid and can be widely performed. Even if PCR detection does not allow discrimination between infection and contamination by non‐germinated spores present in the digestive tract (Gisder et al., 2020), it can reliably detect even low‐intensity or latent infections (Graystock et al., 2015). Hence, PCR methods are suitable for screening and could provide detailed insights into the role of urbanization in pollinator epidemiology and pollinator‐parasite interactions (Cohen et al., 2022).

In this study, we used a molecular approach to investigate the impact of urban green areas in shaping the occurrence of parasites in two bumblebee species, namely *Bombus terrestris* (Linnaeus, 1758) and *Bombus pascuorum* (Scopoli, 1763) both largely abundant also in cities (Tommasi, Pioltelli, et al., 2022). We focused on the parasite richness, indicating the number of different parasite taxa detected in each sample and on the probability of infection thus detecting at least a single parasite in a sample. Even if related to each other, these two variables indicate different aspects: while one indicates the possibility of co‐infection and the parasite load, the other describes how probable it is to be infected by any of the studied parasite species. We considered several urban scales by focusing on the landscape structure, local features and honeybee presence as potential intermediate vectors. Specifically, we evaluated the impact of these features in shaping the occurrence of the commonest parasite known to cause major problems in bumblebees, trypanosomatids (*Crithidia* spp.), microsporidians (*Nosema* spp.) and neogregarines (*Apicystis* spp.) (Cilia et al., 2021; Ivers et al., 2022; Theodorou et al., 2016; Youngsteadt et al., 2015). Since the reduced and fragmented green habitats of the more urbanized landscape are expected to concentrate bees to the remnant patches available for foraging (Dylewski et al., 2019; Quistberg et al., 2016), we hypothesized to observe an increase in the parasite richness and probability of infection in the smaller and more fragmented green habitats of the more urbanized areas. At the local scale, high availability and diversity of floral resources are expected to increase pollinator community richness and abundance (Hülsmann et al., 2015; Tommasi et al., 2021) thus following disease ecology theory (Becker et al., 2015) we hypothesized to also observed positive correlation among flower abundance and parasite richness and probability of infection. Furthermore, based on previous research showing a positive correlation between beekeeping and the spread of parasites to wild species (Graystock, Blane, et al., 2016), we expected a higher parasite richness and probability of infection in bumblebees collected from study sites with a higher abundance of honeybee hives in the surrounding and/or located closer to the apiary.

## 2 MATERIALS AND METHODS

### 2.1 Study species: bumblebees and parasites

As previously stated, two different co‐occurring species of bumblebees have been selected for this study, *B. terrestris* (Linnaeus 1758) and *B. pascuorum* (Scopoli 1763). These are common pollinators in Europe and widely adopted model species with well‐known ecology and biology (Rasmont et al., 2008; Theodorou et al., 2021). These two important wild pollinators are usually found in several contexts that range from natural to urbanized sites (Intoppa et al., 1995; Meeus et al., 2021; Polce et al., 2018; Tommasi, Pioltelli, et al., 2022). Therefore, they are suitable for understanding how wild pollinators are affected by land‐use change and which strategies they adopt to cope with urban stressors (Eggenberger et al., 2019; Theodorou et al., 2021). In addition, these two species have different demands in terms of foraging and nesting habits.

Three different taxa of pathogens known to affect both honeybees and wild bees have been selected, namely microsporidians (Phylum: Rozellomycota), trypanosomatids (Phylum: Euglenozoa) and neogregarines (Phylum: Apicomplexa) parasites. Several parasite species belong to these taxa and are known to affect bumblebees also producing different effects; however, these symbionts similarly lead to an overall reduction in individual lifespan and colony fitness (Botías et al., 2021; Gómez‐Moracho et al., 2020, 2022; Graystock et al., 2013; Larsson, 2007; Otti & Schimid‐Hempel, 2008; Yourth et al., 2008). Since the aim of this work was to describe general patterns of infection distribution in response to landscape features no species‐specific assays on these parasites were performed. Relatively little is known about the epidemiological dynamics of these three groups in wild pollinators; therefore, all bumblebee specimens have been tested for all of them. Among the microsporidians, *Nosema* spp. is known to affect managed and wild bees and is transmitted through oral‐faecal routes (Solter, 2014). Specifically, *Nosema bombi* and *N. ceranae* are the most common species detected both in bumblebees and *Apis mellifera* (Graystock et al., 2013; Higes et al., 2010). Among trypanosomatids, *Crithidia* spp. have been previously detected in wild bee species (Strobl et al., 2019; Yourth et al., 2008). It is a common parasite target of several pieces of research on bumblebees (Imhoof & Schmid‐Hempel, 1999; Plischuk & Lange, 2009); and is transmitted via oral‐faecal routes like *Nosema* spp (Brown et al., 2000). Effects of *Crithidia* spp. vary from the chronic reduction of foraging efficiency to acute increasing mortality (Brown et al., 2000; Gegear et al., 2006). Similarly, the neogregarines are understudied parasites of bees and the only known species detected in honey bees, bumblebees and solitary bees is *Apicystis bombi* (Lipa & Triggiani, 1996; Plischuk et al., 2011; Tian et al., 2018). Bumblebees infected by this species are known to have reduced fat bodies and increased mortality (Graystock, Meeus, et al., 2016).

### 2.2 Study site characterization and sampling

**FIGURE 1:**
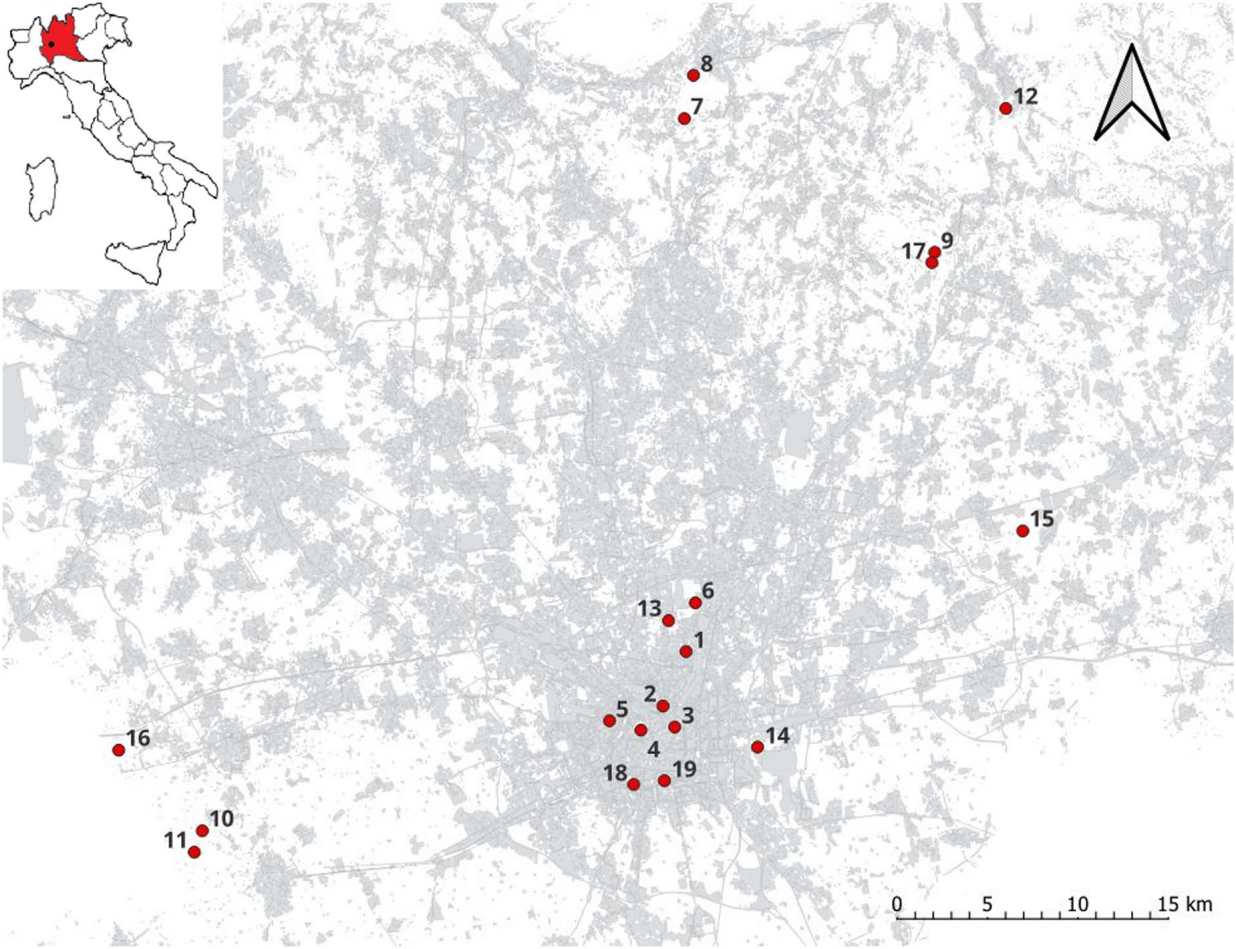
Map reporting the localization of study sites across the urbanization gradient, grey areas correspond to cemented surfaces.

Sampling activities were performed in July 2019 in 19 sites located in an urbanized area in the central portion of north Italy covering four administrative provinces of northern Italy (i.e. Milano, Monza e della Brianza, Lecco and Como) (Figure 1). In order to avoid non‐independence, the distance between sites was at least 2 km, above the foraging range observed for these species (Biella et al., 2022). Sampling sites have been selected to cover a gradient of growing urbanization ranging from semi‐natural areas to the more urbanized context of the city centre of Milan. In semi‐natural areas, the sampling sites were characterized by continuous meadows rich in spontaneous flowers, typically located close to forest patches and far from intensive agriculture conditions. Conversely, in urban areas, the sampling sites were located within urban parks surrounded by an urban matrix, characterized by frequent mowing and lower availability of flowers, mainly represented by ornamental flowerbed. To select sampling sites impervious surfaces (i.e. concrete, building and asphalt) have been mapped using regional land‐use cartography (2018‐DUSAF 6.0; https://www.dati.lombardia.it/Territorio/Dusaf‐6‐0‐Uso‐del‐suolo‐2018/7rae‐fng6) as explained in detail in (Tommasi, Pioltelli, et al., 2022), afterwards sampling sites have been chosen along a visible gradient of impervious cover.

In each site, five to six workers per species were collected with haphazard walks within a 50 m × 50 m plot using an entomological net. After collection samples were stored at −80°C until further analyses. Overall 192 individuals belonging to the investigated bumblebee species (96 *B. pascuorum* and 96 *B. terrestris*) have been subjected to gut DNA extraction and multi‐target parasite screening.

Floral resources were estimated in each site by counting the total number of flowers within six squares 1 m × 1 m placed in flowering spaces within the sampling area (see Tommasi, Biella, et al., 2022; Tommasi, Pioltelli, et al., 2022 for a detailed explanation of floral resources availability estimation). The data were also used to estimate the diversity of the available floral resources in each site and was calculated as Shannon diversity Index (Shannon, 1948). The percentage of green habitat (i.e. meadows, forests and urban green spaces) in the 1 km radius buffer surrounding each sampling site has been calculated using the previously mentioned land‐use map (see Tommasi, Pioltelli, et al., 2022 for a detailed explanation of land‐use categorization). Specifically, the buffer size has been selected according to the maximum foraging range expected for these species (Greenleaf et al., 2007; Knight et al., 2005). Furthermore, green habitat fragmentation has been estimated for each buffer surrounding the sampling sites by calculating the Euclidean nearest neighbour distance (ENN) of green habitat patches through the package *landscape metrics* (Hesselbarth et al., 2019) in the software R (version 2022.12.0).

The distance of each sampling site from the closer honey bee hive and the number of hives in the 1 km buffer surrounding the sites have been calculated through the distance matrix function of QGIS (version 3.28.4). Honeybee hive locations have been obtained from the national beekeeping database (BDA) upon request and released by the competence office of each province involved in the present study. Variables calculated for each sampling site are available in Table S1.

### 2.3 DNA extraction and pathogen detection

To detect endoparasites the gut of each specimen has been extracted using tweezers and sterilized using a 3% bleach solution and 70% ethanol in between dissections to avoid contamination between individuals. Subsequently, the genomic DNA was extracted from gut samples by using Qiagen© DNeasy® PowerSoil Pro Kit (Qiagen) following the manufacturer's protocol. Target DNA fragments, exclusive for the investigated endoparasites, have been amplified through PCR assays. Specific primer pairs were adopted to amplify sequences belonging to 18S region (SSU) for trypanosomatids detection, 16S region (SSU) for microsporidia detection and sequences that include part of 5.8S, section of ITS2 and part of 28S section (SSU) for *A. bombi* detection (see Table 1 for further information and references). WonderTaq® DNA polymerase was used for amplification following the manufacturer's instruction with 5 μL of DNA template. The thermal profile used to perform PCR was the same for all three primer pairs and was organized in 10 pre‐amplification cycles of 30 s at 94°C, 30 s at 60°C and 45 s at 72°C followed by 30 amplification cycles of 30 s at 94°C, 30 s at 57°C and 45 s at 72°C (Graystock et al., 2020).

**TABLE 1:** Primers pairs selected to detect each target endoparasite with relative sequences, target region of rRNA SSU gene and expected amplicon dimension.

Target endoparasites	Primers ID	Target region‐gene	Sequences	Amplicon dimension (bp)	References
Microsporidians (*Nosema* spp).	MSporF2 MSporDegR	16S ‐ SSU	5′‐AGTGGTGCATGGCCGTTTTC‐3′ 5′‐GGTGTGTRCAAAGAACAGGG‐3′	270	Mullins et al. (2020)
Trypanosomatids (*Crithidia* spp.)	CB‐SSUrRNA‐F2 CB18SR2	18S ‐ SSU	5′‐CTTTTGACGAACAACTGCCCTATC‐3′ 5′‐TGCTCCTTTGTTATCCCATGCT‐3′	584	Tripodi et al. (2018)
Neogregarines (*Apicystis bombi*)	Apicyst357F Apicyst357R	Region including part of 5.8S, ITS2 and 28S ‐ SSU	5′‐AGCGATGGATGTCTTGGGTC‐3′ 5′‐CCTAGTTAGTTTCTTTTCCTCCGC‐3′	357	Mullins et al. (2020)

Capillary electrophoresis was performed with Qiagen© QIAxcel® Advanced System (Qiagen), using the Qiagen© QIAxcel® DNA High‐Resolution screening kit (Qiagen) in order to visualize expected band presence and size related to each parasite (see Table 1). A pool of positive samples for each endoparasite is depicted in Figure S1. The amplification of correct target was also confirmed by sequencing three to five amplicons for each primer set.

### 2.4 Statistical analysis

The results of PCR screening were used to classify each specimen as positive or negative to at least one infection and calculate the parasite richness in each specimen (i.e. the number of different parasite targets detected in each sample). Parasite richness and presence/absence of infection were included as dependent variables in different generalized linear mixed models to estimate their relationship with the investigated covariates. The responses of the two species were tested separately and covariates were chosen following our ecological expectation. Specifically, in all the models we included the same covariates namely the percentage of green habitat and its fragmentation (ENN) as well as the abundance and diversity of floral resources. Moreover, each model encompassed the distance from hives and the number of hives in the surroundings. The amount of impervious cover initially included in the models with the other covariates as a descriptor of urbanization, was excluded by the models because of the high collinearity with the percentage of green habitat evaluated through variance inflation factor (VIF) (see also the correlation plot in Figure S2). Presence/absence of infections was used as dependent variables in a Generalized Linear Mixed Model (GLMM) (Magnusson et al., 2017) with a binomial distribution (accounting for binary presence/absence data) to evaluate changes in the probability of being infected in response to the mentioned independent variables. Changes in parasite species richness per sample in response to the considered covariates have been evaluated through GLMM with Poisson distribution (accounting to count data), in both the models sampling sites were included as a random effect. Final models were obtained through a backward stepwise model selection approach based on AIC (Zuur et al., 2009). Data analysis was performed using R (version 3.6.1).

## 3 RESULTS

The three parasite groups were detected in both the bumblebee species with evident differences in terms of infection rate. Infection of *Microsporidia* occurred in similar rates in *B. pascuorum* and *B. terrestris* (42.71% in *B. pascuorum* and 46.88% in *B. terrestris*), while different patterns of infections were detected in the case of trypanosomatid (8.33% in *B. pascuorum* and 46.88% in *B. terrestris*) and neogregarines (1.04% in *B. pascuorum* and 15.63% in *B. terrestris*).

The combination of coinfection rates highlighted different coinfection patterns in the two bumblebee species (Table 2), with *B. terrestris* hosting a higher parasite richness compared to *B. pascuorum*. In particular, *B. pascuorum* showed a 51.04% rate of carrying 0 infections, while *B. terrestris* demonstrated a much lower probability (21.88%). Furthermore, 78.13% of *B. terrestris* carried at least one parasite, while in *B. pascuorum* it was 48.96%.

**TABLE 2:** Coinfection rates in both bumblebee species.

Coinfection combination	*Bombus pascuorum* (%)	*Bombus terrestris* (%)
Trypanosomatids (*Crithidia* spp.) + Neogregarines (*Apicystis bombi*)	0	8.33
Microsporidians (*Nosema* spp.) + Neogregarines (*Apicystis bombi*)	0	8.33
Microsporidians (*Nosema* spp.) + Trypanosomatids (*Crithidia* spp.)	4.33	19.79
All three parasites	0	3.13

*Note*: Each line contains a combination of different endoparasite coinfection and their occurrence frequency in all our specimens divided for species. Each box contains the sum of the relative frequency of each coinfection combination in our samples expressed as percentage.

**FIGURE 2:**
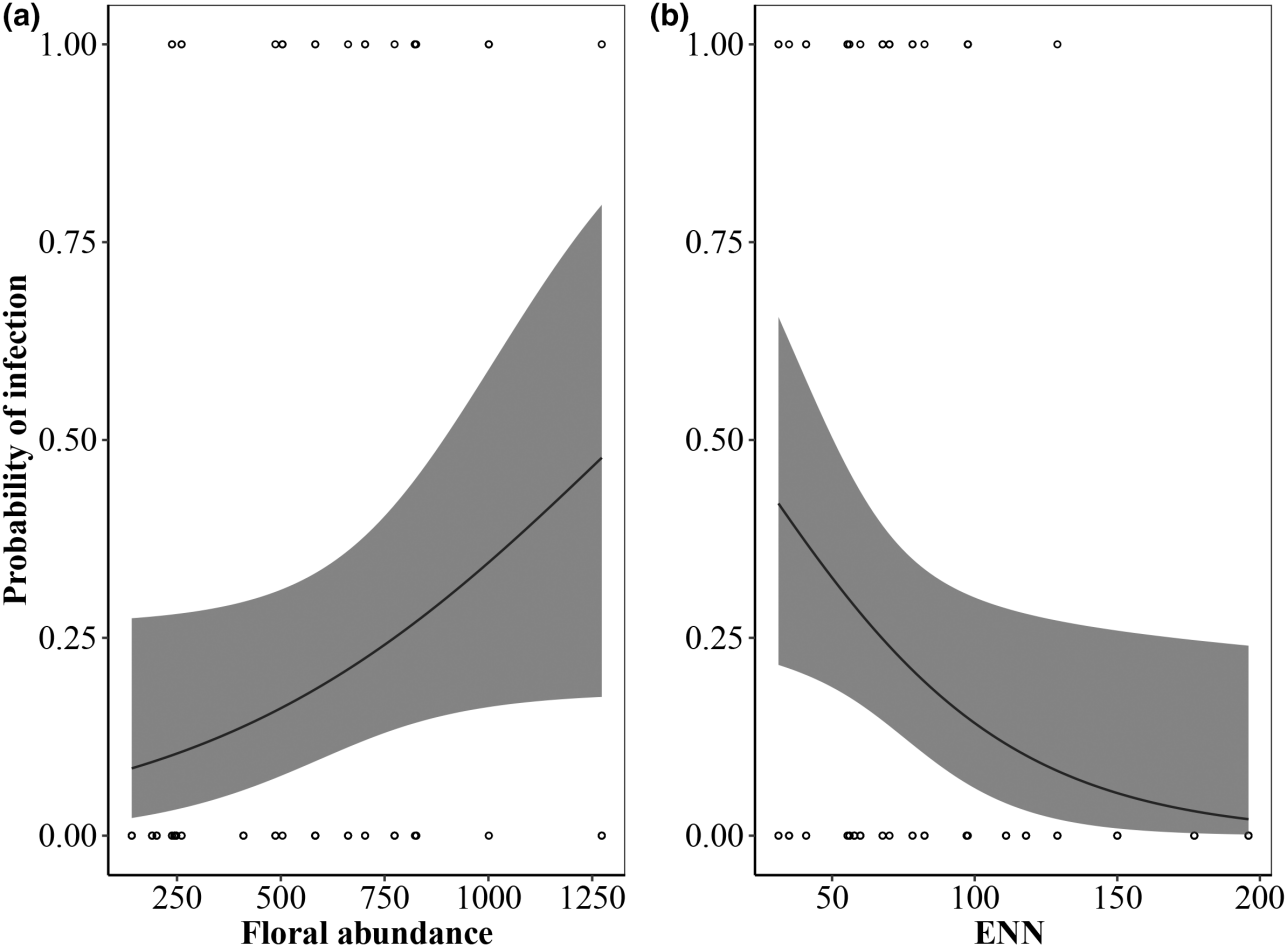
Probability of infection (estimated from presence/absence of infections per specimen) as a function of (a) floral resource abundance and (b) Euclidean nearest neighbour distance (ENN) in *Bombus pascuorum*. The black line and grey areas indicate the relationship and its confidence intervals (*α* = 95%) estimated through generalized linear mixed models.

The two bumblebee species responded differently to the factors describing the urbanization phenomenon. The probability of infection significantly increased with floral abundance (Figure 2a, Table 3) and decreased with ENN (Figure 2b, Table 3) in *B. pascuorum*. All the other predictor variables (i.e. percentage of green habitat, distance from hives, floral diversity and the number of hives in the surrounding of sites) did not show significant effects on parasite richness (Table 3). However, none influenced *B. terrestris*' probability of infection, as it was not influenced by the evaluated predictor variables.

**TABLE 3:** Output of generalized linear mixed models of parasite richness and probability of infection as a function of the candidate covariates supposed to shape parasite detection.

Species	Response variable	Full model covariates	Final model covariates	ΔAIC	*χ* ^2^; df	*β*_*i*_	*p*‐value
*Bombus pascuorum*	Probability of infection	Percentage of green habitat	Floral abundance	7.563	5.107; 1	1.22	**0.038**
		Number of hives	ENN		5.871; 1	−0.02	**0.042**
		Distance from hives	(1|site)				
		Floral abundance					
		Floral diversity					
		ENN					
		(1|site)					
*Bombus terrestris*	Probability of infection	Percentage of green habitat	Intercept	5.848	127.95; 1	0.556	**0.009**
		Number of hives					
		Distance from hives					
		Floral abundance					
		Floral diversity					
		ENN					
		(1|site)					
*B. pascuorum*	Parasite richness	Percentage of green habitat	Floral abundance	7.325	4.75; 1	0.919	**0.04**
		Number of hives	ENN		4.428; 1	−0.014	0.073
		Distance from hives	(1|site)				
		Floral abundance					
		Floral diversity					
		ENN					
		(1|site)					
*B. terrestris*	Parasite richness	Percentage of green habitat	Distance from hives	5.823	2.27; 1	0.0004	**0.045**
		Number of hives	Floral abundance		2.359; 1	0.001	0.085
		Distance from hives	(1|site)				
		Floral abundance					
		Floral diversity					
		ENN					
		(1|site)					

*Note*: Final models were obtained through backward stepwise selection using AIC criterion. Differences in the AIC values between full and final models are reported in ΔAIC. Statistical details refer to the final models and are regression coefficient (*β*_*i*_), chi‐square values (*χ* ^2^) and degrees of freedom (df). Significant *p*‐values are reported in bold.

**FIGURE 3:**
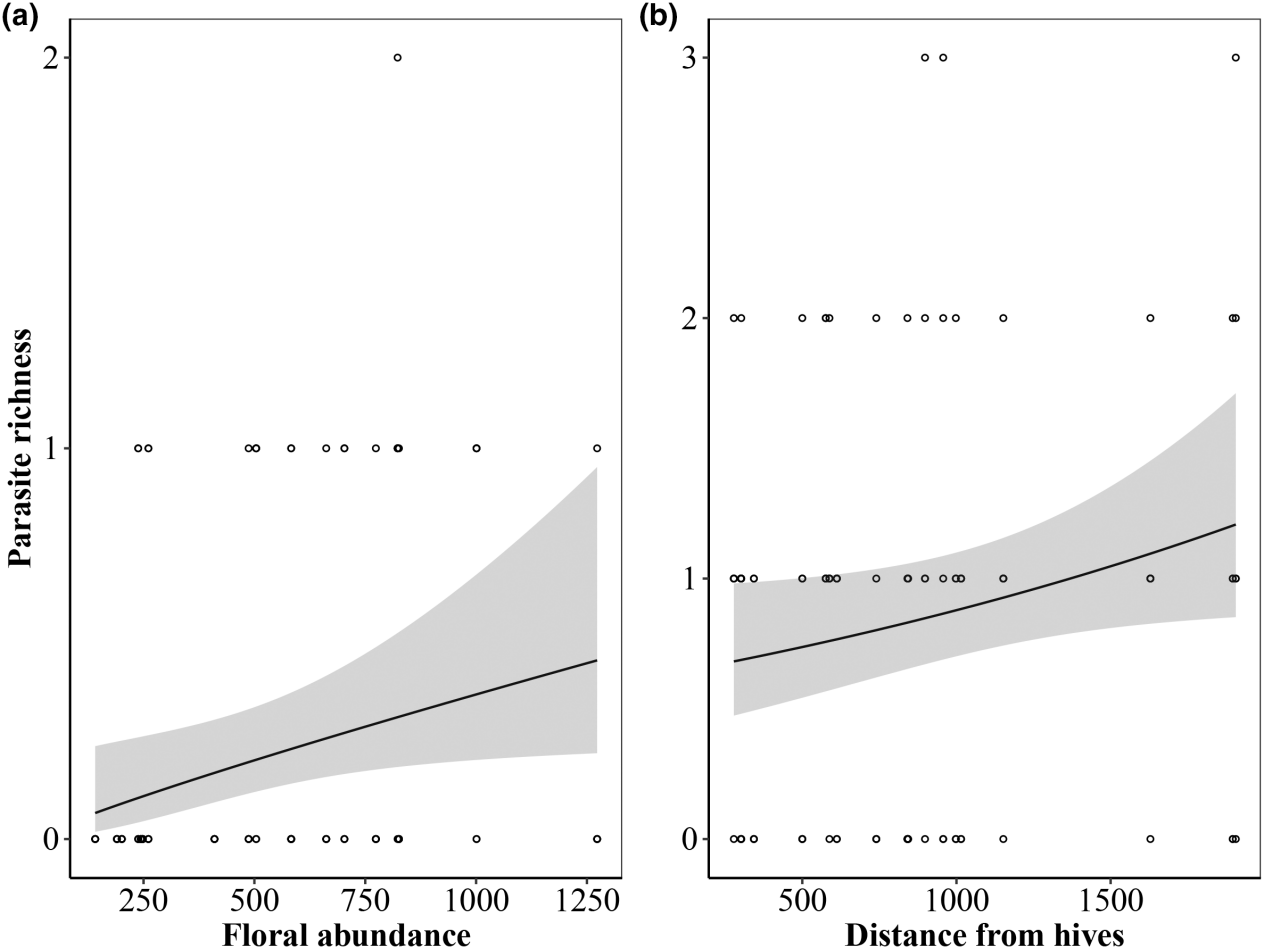
Parasite richness (number of different parasite targets detected in each sample) as a function of (a) floral resource abundance in *Bombus pascuorum* and (b) distance from honeybee hives in *Bombus terrestris*. The black line and grey areas indicate the relationship and its confidence intervals (*α* = 95%) estimated through generalized linear mixed models.

Parasite richness was lower in *B. pascuorum*, with a maximum of 2 target parasites per sample, compared to *B. terrestris* whose maximum was 3 target parasites. Nevertheless, local variables such as the floral abundance and the distance from honeybee hives significantly shaped parasite richness. Specifically, the parasite richness increased with floral abundance in *B. pascuorum* (Figure 3a, Table 3) and distance from honeybee hives in *B. terrestris* (Figure 3b, Table 3). All the other predictor variables (i.e. percentage of green habitat, ENN, floral diversity and the number of hives in the surrounding of sites) did not show significant effects on parasite richness (Table 3).

## 4 DISCUSSION

In this study, we explored urban pollinator‐parasite interactions focusing on the relationships between parasites' incidence and coinfection rate with the green habitat availability and fragmentation, floral resources availability and the proximity to beehives.

Our results showed clear differences in the infection and co‐infection rate in the two bumblebee species, with *B. pascuorum* emerging as less prone to host parasites compared to *B. terrestris*. These differences are largely due to trypanosomatids (*Crithidia* spp.) and neogregarines (*Apicystis* spp.) that were detected with higher rates in *B. terrestris*. Moreover, coinfections due to two or more target parasites were extremely rare in *B. pascuorum* but relatively common in *B. terrestris*. A higher parasite prevalence in *B. terrestris* compared to *B. pascuorum* and other congenerics has been previously reported (Cameron et al., 2011; Goulson et al., 2012; Jabal Uriel et al., 2017) but a reliable explanation of these interspecific differences has not been provided yet. In this context, a number of factors could be involved and constitute valid research questions to be further addressed. The first factor could be that the lower occurrence of infected individuals in *B. pascuorum* may suggest a lack of tolerance of this species toward infections that could significantly reduce the survival of infected individuals and thus the possibility to collect and analyse infected specimens (as we collected only living individuals in this study). On the contrary, *B. pascuorum* may be particularly resistant towards parasite contamination due to morphological, physiological or ecological aspects (e.g. its nesting and foraging habits). Large body size and foraging breath are bee traits supposed to increase exposure to parasites (Cohen et al., 2021), and this could explain the observed idiosyncratic pattern of infection between the two investigated bumblebee species, with *B. pascuorum* being smaller and with a slightly narrower foraging breath (Harder, 1985). Furthermore, the two bumblebees also differ in colony size, where *B. terrestris* and *B. pascuorum* could reach 1000 and 150 individuals per colony, respectively (Von Hagen & Aichhorn, 2014) and this might mediate their epidemiology via intra‐colony transmission.

Based on our results, the landscape green habitat fragmentation significantly shapes parasite occurrence, and in particular, it seems to reduce the probability of infection in *B. pascuorum*. This finding does not support our expectation of having higher parasite occurrence in the more fragmented habitats due to the aggregation of bumblebee hosts in the few green remnants available (amplification effect, Becker et al., 2015). In an urban context, the dispersion of green areas and the presence of inhospitable surfaces (concrete) affects the incidence of bumblebees as they could hardly arrive and forage in progressively isolated green areas. In this case, a lower host availability could explain the observed reduction in parasite occurrence. Therefore, future investigation will benefit from a clear determination of the community of flower visitors in terms of species richness and abundance. This will allow a deeper comprehension of the indirect effects of land‐use features mediated by dilution, thus lower parasite prevalence due to higher species richness and diversity (Civitello et al., 2015), or amplification effects.

The dependency between parasite abundance and host distribution in the urban landscape may result from a major importance of floral resource availability in increasing parasite occurrence, as resulted from our analyses. When flowers are highly available and a rich pollinator community is present there, it is expected to observe a dilution effect of parasites in several hosts, especially over large surfaces (Piot et al., 2019). However, our results may support the alternative hypothesis that floral resources improve parasite transmission due to the higher attractiveness of floral spots and the consequent higher bee aggregation. In this context, both the probability of infection and the parasite richness shown by *B. pascuorum* were higher where more flowers were available for foraging. This result supports the well‐known role of flowers as hubs for parasites spread among individuals (Pinilla‐Gallego et al., 2022). Indeed, about 10% of flowers were found to host one or more parasites of bees (Graystock et al., 2020) and shorter and wider flowers promote higher transmillability (Pinilla‐Gallego et al., 2022). This could be a peculiarity of ‘poor‐quality’, fragmented landscapes and of disconnected urban green areas, where flowers occur in aggregated ways or cover specific areas, thus aggregating pollinators too and promoting infection, as higher bee pathogens are found in flower strips near isolated semi‐natural patches (Piot et al., 2019). This highlights potential risks associated with those interventions that aim at safeguarding pollinators through floral strip planting since these could facilitate the spread of infections in wild bee communities. While in *B. pascuorum* the probability of infection and parasite richness were shaped by landscape and local features a similar trend was not highlighted in *B. terrestris* which resulted in more susceptible to parasite infection independently from landscape and local features.

Interestingly, *B. terrestris* parasite richness was affected by the proximity to beekeeping sites. Other studies highlighted a major role of apiculture for the spread of parasites (Martínez‐López et al., 2021; Nanetti et al., 2021) but without focusing on beekeeping activities within the urban environment. This will require further insights since the worry of pollinator decline is pushing many citizens and companies to adopt honeybee hives in cities (Sponsler & Bratman, 2021). In this study, parasite richness decreased in proximity to honeybee hives, likely because apiculture disproportionally increases the abundance of *Apis mellifera* in the city and thus favouring the spread of parasites on the most abundant host rather than on alternative bumblebee hosts. However, here we evaluated the impact of apiculture using proxy variables of honeybee presence, that do not exhaustively describe the local abundance of *A. mellifera* and do not consider beekeeping practices that are known to shape parasite transmission to wild species (Piot et al., 2022). A detailed estimation of these variables would improve the comprehension of the impact of apiculture on the spread of parasites to urban and non‐urban wild bees.

## 5 CONCLUSIONS

Landscape and local features of urban green habitats shaped the occurrence of parasites with marked differences among the two investigated bumblebee species. This novel finding highlights the importance of designing proper target conservation efforts based on pollinator species‐specific knowledge. The planting of flower strips is gaining importance among the efforts to safeguard pollinators; however, our findings also shed light on the potential detrimental effects of these practices that must be considered by public administrators. Further investigation related to the spatial arrangement of flower patches, as well as an evaluation of the most suitable flower species in terms of morphological traits and shapes with lower potential for parasite transmission will be useful to refine these conservation measures. Moreover, we recommend considering the configuration of green areas at the landscape scale, here confirmed as a driver able to shape parasite dynamics, in implementing these conservation measures. The results obtained here will contribute to the fine‐tuning of the interventions aimed at improving pollinator health and well‐being also in urban areas, indirectly contributing to our well‐being since as resumed by the one‐health concept human and ecosystem health are inextricably linked.

## AUTHOR CONTRIBUTIONS

**Nicola Tommasi:** Conceptualization (lead); data curation (lead); formal analysis (lead); investigation (lead); methodology (lead); supervision (lead); writing – original draft (lead). **Beatrice Colombo:** Formal analysis (equal); investigation (equal); writing – original draft (equal). **Emiliano Pioltelli:** Writing – review and editing (equal). **Paolo Biella:** Funding acquisition (equal); writing – review and editing (equal). **Maurizio Casiraghi:** Writing – review and editing (equal). **Andrea Galimberti:** Funding acquisition (equal); writing – review and editing (equal).

## BENEFIT‐SHARING STATEMENT

Benefits from this research arise from the sharing of our data and results on public databases as described above.

## Supporting information


Appendix S1

## Data Availability

All relevant data are within the paper or stored in figshare at the following link https://figshare.com/s/c58df945039de5747072.
