# Supplementary material for: Urban habitat fragmentation and floral resources shape the occurrence of gut parasites in two bumblebee species
Source: Ecol Evol. 2023 Jul 12;13(7):e10299. doi: 10.1002/ece3.10299 (PMC10338672; doi:10.1002/ece3.10299)
Supplement: Supplementary file 1 — Appendix S1 [file ECE3-13-e10299-s001.docx]

Urban habitat fragmentation and floral resources shape the occurrence of gut parasites in two bumblebee species

Nicola Tommasi^1,2^, Beatrice Colombo^1^, Emiliano Pioltelli^1,2^, Paolo Biella^1,2^, Maurizio Casiraghi^1,2^, Andrea Galimberti^1,2^

1- Department of Biotechnology and Biosciences, ZooplantLab, University of Milano-Bicocca, Milan, Italy

2 - NBFC, National Biodiversity Future Center, Palermo 90133, Italy

**Correspondence**:

Nicola Tommasi

nicola.tommasi@unimib.it

**TABLE OF CONTENT**

**Pag 2:** Table S1 - Variables distribution among sampling sites

**Pag 3:** Figure S1 - Results of diagnostic PCR obtained through capillary electrophoresis.

**Pag 4**: Figure S2 - Variables correlation plot

**Table S1- Variables distribution among sampling sites.** Site ID refers to the same identification number used in figure 1. The percentage of green habitat is calculated starting from the coverage of green habitat in a 1 km buffer surrounding each sampling site. Floral abundance is estimated through field evaluation of the number of flowers present in six quadrats 1 m × 1 m within the study plots. Number of hives refers to the total number of honeybee hives within a 1 km buffer surrounding each sampling site while distance from hives refers to the distance between the sampling sites and closest hive. Floral diversity refers to the Shannon diversity index calculated for each site starting from vegetational data collected in six quadrats 1 m × 1 m within the study plots. Nearest neighbor distance (ENN) is the green habitat fragmentation index calculated within a 1 km buffer surrounding each sampling site.

| **Site ID** | **Percentage of green habitat** | **Floral abundance** | **N° of hives** | **Distance from hives (m)** | **Floral diversity** | **ENN** |
| --- | --- | --- | --- | --- | --- | --- |
| 1 | 7.62 | 826 | 2 | 343 | 1.095 | 129 |
| 2 | 5.34 | 410 | 0 | 1152 | 1.52 | 150 |
| 3 | 10.84 | 244 | 0 | 1896 | 1.32 | 111 |
| 4 | 15.64 | 583 | 4 | 957 | 1.33 | 78.2 |
| 5 | 7.82 | 583 | 0 | 1906 | 1.39 | 177 |
| 6 | 38.42 | 201 | 1 | 998 | 1.513 | 97.2 |
| 7 | 55.74 | 238 | 70 | 612 | 1.4963 | 31.2 |
| 8 | 52.00 | 774 | 58 | 500 | 1.5162 | 59.9 |
| 9 | 78.00 | 703 | 134 | 588 | 1.72 | 40.9 |
| 10 | 35.35 | 1001 | 0 | 1015 | 0.1564 | 70.1 |
| 11 | 37.74 | 141 | 35 | 740 | 1.189 | 196 |
| 12 | 39.99 | 504 | 20 | 843 | 1.65 | 55.4 |
| 13 | 38.34 | 248 | 11 | 841 | 0.9917 | 57.8 |
| 14 | 25.26 | 1273 | 60 | 576 | 1.344 | 82.4 |
| 15 | 28.84 | 261 | 94 | 301 | 1.812 | 67.7 |
| 16 | 51.02 | 823 | 20 | 898 | 1.642 | 97.6 |
| 17 | 59.08 | 487 | 148 | 302 | 1.55 | 34.9 |
| 18 | 10.04 | 662 | 6 | 278 | 2.285 | 56.1 |
| 19 | 10.33 | 190 | 0 | 1629 | 0.4485 | 118 |

**Figure S1**. Pool of positive samples. a*) B. terrestris* sample positive for Apicystis bombi; b) *B. terrestris* sample positive for microsporidians (Nosema spp.); c) *B. terrestris* positive sample for trypanosomatids (Crithidia spp.); d) Negative control.


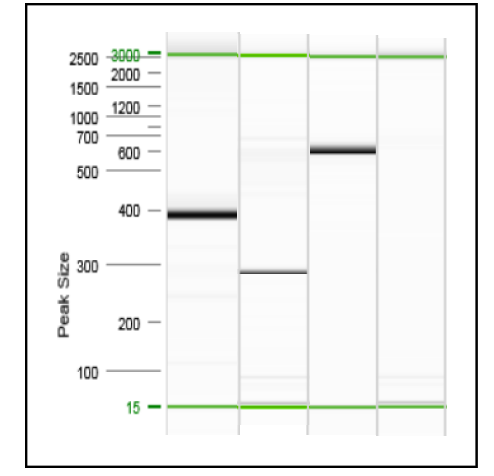


**Figure S2- Variables correlation plot**

Graphical display of correlation matrix obtained using the r package “corrplot” (Friendly, 2022).Positive correlations are represented in blue while negative correlation in red. The size and color of dots are proportional to correlation coefficients.

**
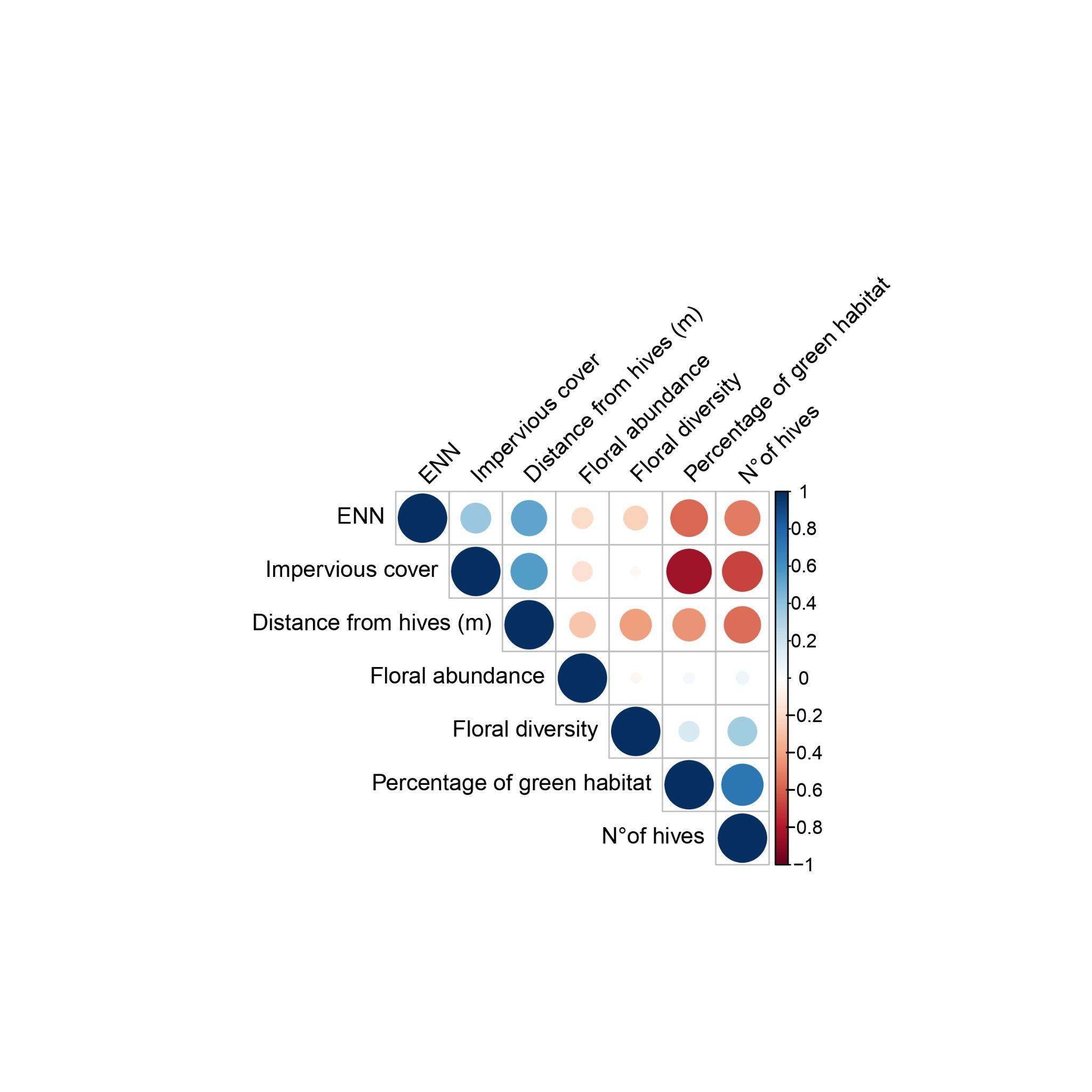
**

**REFERENCE**

Michael Friendly (2002). Corrgrams: Exploratory displays for correlation matrices. The American Statistician, 56, 316–324.
